# Nonionic Surfactants can Modify the Thermal Stability of Globular and Membrane Proteins Interfering with the Thermal Proteome Profiling Principles to Identify Protein Targets

**DOI:** 10.1021/acs.analchem.2c04500

**Published:** 2023-02-13

**Authors:** Emmanuel Berlin, Veronica Lizano-Fallas, Ana Carrasco del Amor, Olatz Fresnedo, Susana Cristobal

**Affiliations:** † Department of Biomedical and Clinical Sciences, Cell Biology, Faculty of Medicine, Linköping University, Linköping 581 85, Sweden; ‡ Department of Physiology, Faculty of Medicine, and Nursing, University of the Basque Country UPV/EHU, Leioa 489 40, Spain; § Ikerbasque, Basque Foundation for Sciences, Department of Physiology, Faculty of Medicine, and Nursing, University of the Basque Country UPV/EHU, Leioa 489 40, Spain

## Abstract

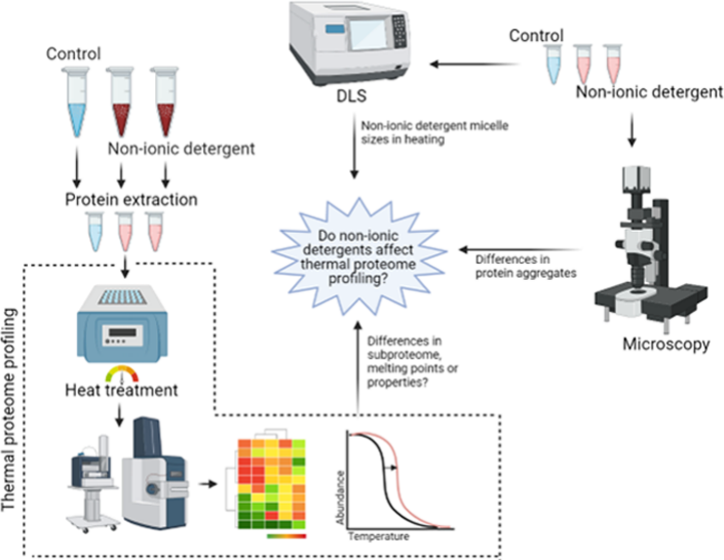

The membrane proteins are essential targets for understanding cellular function. The unbiased identification of membrane protein targets is still the bottleneck for a system-level understanding of cellular response to stimuli or perturbations. It has been suggested to enrich the soluble proteome with membrane proteins by introducing nonionic surfactants in the solubilization solution. This strategy aimed to simultaneously identify the globular and membrane protein targets by thermal proteome profiling principles. However, the thermal shift assay would surpass the cloud point temperature from the nonionic surfactants frequently utilized for membrane protein solubilization. It is expected that around the cloud point temperature, the surfactant micelles would suffer structural modifications altering protein solubility. Here, we show that the presence of nonionic surfactants can alter protein thermal stability from a mixed, globular, and membrane proteome. In the presence of surfactant micelles, the changes in protein solubility analyzed after the thermal shift assay was affected by the thermally dependent modification of the micellar size and its interaction with proteins. We demonstrate that the introduction of nonionic surfactants for the solubilization of membrane proteins is not compatible with the principles of target identification by thermal proteome profiling methodologies. Our results lead to exploring thermally independent strategies for membrane protein solubilization to assure confident membrane protein target identification. The proteome-wide thermal shift methods have already shown their capability to elucidate mechanisms of action from pharma, biomedicine, analytical chemistry, or toxicology, and finding strategies, free from surfactants, to identify membrane protein targets would be the next challenge.

## Introduction

Understanding the consequence of the interaction of membrane proteins with small molecules such as drugs or chemicals would require high-throughput methodologies supporting a rapid screening of several thousand protein candidates from any cell type. The membrane proteins are essential targets for understanding cellular function. They are key nodes that regulate cell communication and initiation of signal transduction pathways. Membrane proteins are largely represented among the drug targets for their capability to orchestrate cellular responses.^1^ Moreover, the membrane proteins are primary interactors with small chemicals present in extracellular compartments, could facilitate the cellular uptake,^2^ and could be responsible for the molecular initiation events leading to adverse outcome pathways for human health.^3^

Different methodologies have been applied for membrane protein target identification. In general, there were methods with low throughput capability and a sufficient similarity to previously described targets as a prerequisite for new findings.^1^ Important changes in the field of target engagement arise with the introduction of the protein thermal shift assay in a proteome-wide context. The Cellular Thermal Shift Assay relayed on a large collection of antibodies for protein identification and quantitation.^4^ However, the introduction of mass spectrometry (MS) to analyze the thermally induced changes of proteome solubility was pivotal to enable the unbiased identification of targets from soluble proteome in methods called Thermal Proteome Profiling (TPP)^5^ and later Proteome Integral Solubility Alteration Assay.^5,6^ The developments of these methodological approaches have recently explored many scientific problems defined by interactions of small chemicals and cellular proteins from biomedicine and analytical chemistry.^7−104^ At our lab, we have focused on implementations for its application to biodiscovery^8^ or toxicology.^9^

Searching for targets in proteomes containing globular proteins facilitates the application of this methodology because the protein–chemical interaction is the main or only stimulus that induces perturbation in protein solubility under the thermal treatment. The factors affecting and ruling protein solubility enlarge when the studied proteome contains a mixture of globular and membrane proteins. Therefore, the next challenge was how to implement these methods for the identification of membrane protein targets. The reported strategy aims to enlarge the soluble proteome with the addition of nonionic surfactants to the solubilization buffer. The surfactants tested are routinely used in membrane protein studies, such as nonyl-phenyl-poly(ethylene glycol) (NP-40S)^10^ or Igepal CA-630 (Igepal), composed of octyl-phenoxy(polyoxyethylene)ethanol.^11^

These nonionic surfactants are substitutes for the original octyl-phenoxy(polyoxyethylene)ethanol (NP-40) that, although it has been widely used, is no longer produced. The chemical structure of Igepal resembles the original NP-40 formulation better than the new NP-40S. However, NP-40S offers closer values to NP-40 in parameters that are important for micelle formation, such as hydrophile–lipophile balance or molecular weight.^12^ The cloud point temperature (CPT) is one of the most distinct physical properties of nonionic surfactants. It is the temperature above which a micellar solution of nonionic surfactant spontaneously forms a two-phase separation. The aqueous micellar two-phase system has been frequently used for the fractionation of proteins with different hydrophobicity.^13^ For NP-40 and NP-40S, the CPT occurs at 45–50 and 53–67 °C for Igepal. Looking into the TPP workflow for the identification of membrane proteins, it is important to observe that the thermal shift spans from 37 to 67 °C. This temperature range includes the specific CPTs of the nonionic surfactants used to increase the membrane protein solubilization.^10^

The expected temperature-dependent modifications of the solution with micelles of nonionic surfactants would include at least micellar stratification.^14^ Additionally, during the increased temperature and achievement of CPT, the surfactant micelles are dehydrated, acquiring an enlarged and elongated shape.^15^ Over the CPT, it was reported that micelle-embedded proteins would be oversaturated in the micelle-rich layer while non-micelle-bound proteins exist in the aqueous-rich layer.^16^ This may cause crowding, protein–protein interactions, micelle–protein interactions, micelle aggregation, and diverse changes in the local environment of the proteome, which may affect protein stability.^17,105^

Given the numerous studies based on the solubilization of membrane proteins with nonionic surfactants, it has been assumed that the membrane proteome in micelles of nonionic surfactants could be compatible with the TPP principles.^10^ The TPP principles for the identification of protein targets are solely based on unique alterations in protein solubility, specifically, the beneficial effects of protein–chemical interactions to increase protein thermal tolerance.^5^ Therefore, insufficient attention has been paid to evaluating the modification of the aqueous micellar solution at a temperature close to or over surfactant CPT and if the structural and density changes on the solvent solution could contribute to altering the protein stability in solution independently of any protein–chemical interaction. This evaluation would offer new insights to define if the chemical–protein interaction in solubility is still a robust parameter to identify membrane protein targets in a solution containing nonionic surfactant micelles that are very malleable within the thermal shift range of temperatures.

In this study, we evaluate the dynamics of nonionic surfactant micelles along with the thermal shift methodology and evaluate the temperature-dependent alteration of the solubility of the soluble and membrane proteome. The interactions of nonionic surfactants with the soluble and membrane proteome could alter their thermal stability interfering with the proteome-wide thermal shift principles. Considering the urgency to offer high-through methodologies for the unbiased identification of membrane protein targets, this study aims to provide the factors and constraints for the future implementation of TPP for the identification of membrane proteins.

## Materials and Methods

### Collection of Liver Tissue

Livers from 2-month-old female Sprague Dawley rats were obtained from the University of the Basque Country with an ethics approval code of M20/2016/237. The procedures conducted on the animals were approved by the Ethics Committee for Animal Welfare of the University of the Basque Country UPV/EHU and followed the EU Directives for animal experimentation.

### Nonionic Surfactants in the Study

Octyl-phenoxy(polyoxyethylene)ethanol, named Igepal CA-630 (Igepal), and nonyl-phenyl-poly(ethylene glycol), named Nonidet-P40 substitute (NP-40S).

### Soluble Proteome

Liver tissue was resuspended in buffer containing PBS (control), 0.4% (v/v) Igepal, or 0.4% (v/v) NP-40S in PBS and solubilized.^10^ The insoluble fraction was sedimented by centrifugation at 100,000*g* for 60 min at 4 °C.^8^ The soluble proteome was used to perform the thermal proteome profiling assay. The pellets were lysed with 100 μL of RIPA buffer (RIPA Lysis Buffer, BOSTER) to perform protein identification and quantification of the insoluble fraction. Protein concentration was determined by the BCA assay.^18^

### Thermal Shift Assay (TSA)

The soluble fractions of the three conditions (control, Igepal, and NP-40S) were heated by duplicate at the specific ten temperatures selected for the thermal shift assay: 37, 42, 46, 49, 51, 53, 55, 58, 62, and 67 °C. Aliquots containing 50 μg of protein were independently heated at the corresponding temperature for 3 min, followed by 3 min at room temperature using a thermocycler (MJ Mini Personal Thermal Cycler PTC 1148, Bio-Rad). Samples were centrifugated at 100,000*g* for 20 min at 4 °C.

### Mass Spectrometry

The samples were reconstituted with 0.1% formic acid in ultra-pure milli-Q water and separated using an EASY nLC 1200 system (Thermo Scientific). Peptides were injected into a precolumn (Acclaim PepMap 100 Å, 75 μm × 2 cm) and separated on an EASY-Spray C18 reversed-phase nano LC column (PepMap RSLC C18, 2 μm, 100 Å, 75 μm × 25 cm). This was applied for a period of 78 min, followed by 40% buffer B against buffer A for 95 min; chromatographic gradients and MS settings are available in the Supporting Information File.

### Peptide and Protein Identification and Quantification

The proteins were identified using Proteome Discoverer (version 2.1, Thermo Fisher Scientific). The MS/MS spectra (raw files) were searched by Sequest HT against the*Rattus norvegicus* database from UniProt (UP000002494; 47,954 entries). A maximum of 2 tryptic cleavages were allowed, and the precursor and fragment mass tolerance were 10 ppm and 0.02 Da, respectively. Peptides with a false discovery rate (FDR) of less than 0.01 and validation based on the *q*-value were used as identified. The minimum peptide length considered was 6, and the FDR was set to 0.1. Proteins were quantified using the average of the top three peptide MS1 areas, yielding raw protein abundances. Common contaminants like human keratin and bovine trypsin were also included in the database during the searches to minimize false identifications. The mass spectrometry proteomics data have been deposited to the ProteomeXchange Consortium via the PRIDE partner repository with the dataset identifier PXD037153.^19^

### Protein Localization

The bioinformatics tool PANTHER,^20^ which utilizes Gene Ontology classification, was used for protein localization. Note that one protein may have several sublocalizations. This selection was made by choosing proteins tagged with the GO term: GO:0016020.

### Dynamic Light Scattering (DLS)

Solutions were PBS as control, 0.4% Igepal, and 0.4% NP-40S. The temperatures chosen were those assayed in the TSA: 37, 42, 46, 49, 51, 53, 55, 58, 62, and 67 °C. To reduce condensation, a silicon solution was added on top of each well. The DLS (DynPro II Platereader, Wyatt) was set to increase the plate temperature following the TSA protocol, and for each temperature, 10 acquisition times (s) were performed. Images were taken between the temperatures 37 and 49 °C since temperatures above were too high for the camera to operate safely. During this thermal exposure, both readings of micelle size and images were taken. Another DLS experiment was performed to measure changes in the DLS measurements from samples heated at 55 and 67 °C (CPTs) and then cooled to 21 °C, like the TPP method that includes the cooling step at RT for 3 min before ultracentrifugation. All experiments were performed in triplicate.

### Microscopy

Visualization of samples exposed to heat was performed to detect surfactant and/or protein aggregates by preparing the samples following the TPP method. Control, Igepal, and NP-40S conditions in a volume of 210 μL each were subjected to 67 °C for 3 min in a thermocycler. This temperature was set to ensure that both, Igepal and NP-40S, samples have passed their CPT. After 3 min of heating, 200 μL of each sample was put into a glass bottom plate (P35-G-1.5-10-C, MatTek), and a cover glass was laid on top of the well. A Leica DMi9 microscope was used with a mounted heating box set at 51 °C (maximum temperature of the heating box) to slow down the cooling of the sample. Visualization was performed within the first 3 min using an HC PL APO CS2 63x/1.20 WATER UV objective with a TL-DIC contrast for 10 ms exposure for each image.

## Results and Discussion

### Solubilization of Membrane Proteins for TPP Analysis

**Figure 1:**
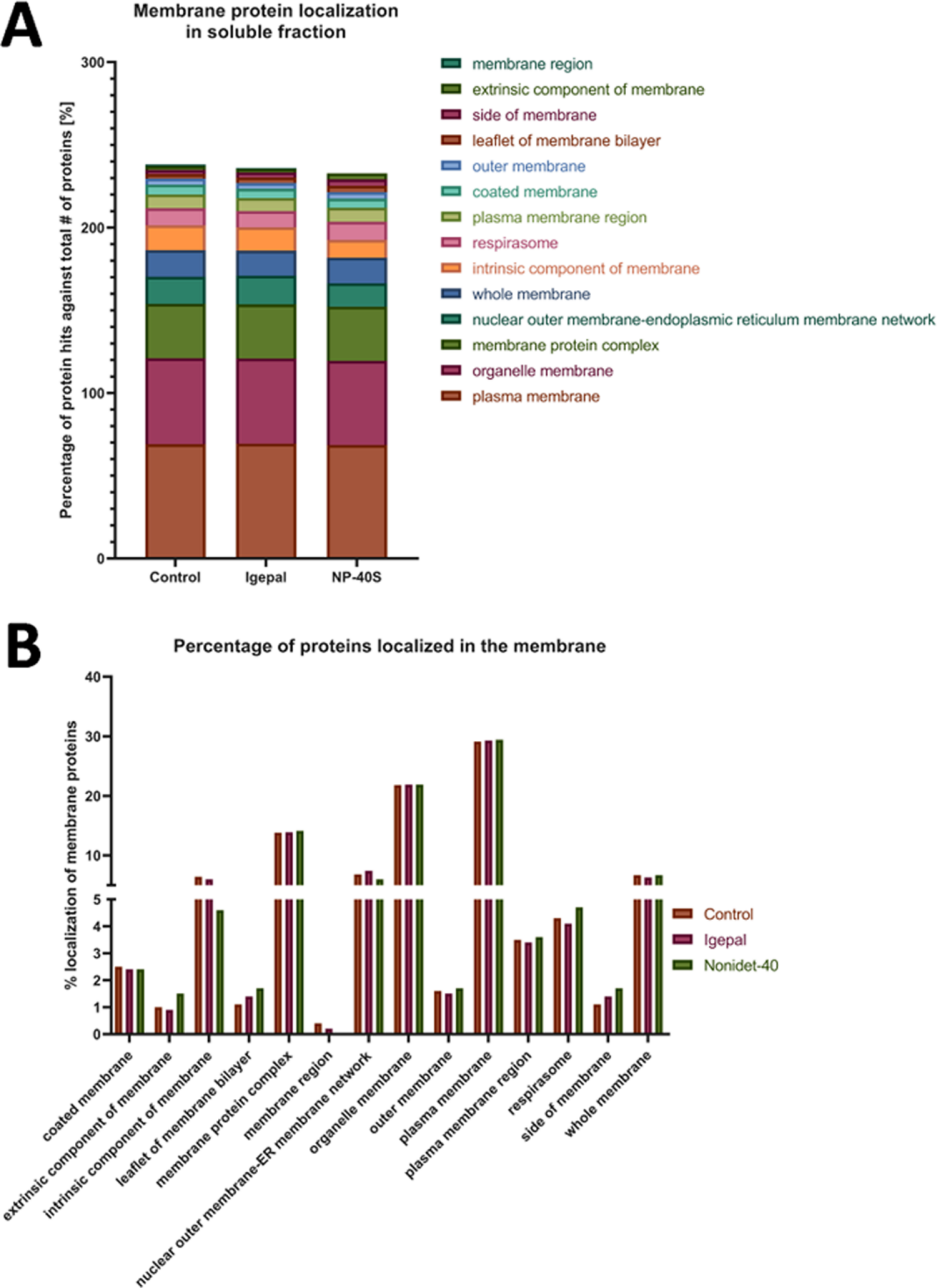
Analysis of the distribution of membrane proteins in the soluble fractions after solubilization with different extraction solutions. (A) Bar diagram representing the distribution of the membrane proteins in the soluble fraction based on GO categories and (B) bar diagram representing changes in the distribution of membrane proteins in different subcellular locations. The extraction solutions were control PBS, 0.4% (v/v) Igepal in PBS, and 0.4% (v/v) NP-40S in PBS. The fractions were analyzed by quantitative mass spectrometry (nLC-MS/MS). Data were classified via PANTHER,^20^ following gene ontology classification.

The introduction of nonionic surfactants, Igepal or NP-40S,^10^ in the extraction solution aims to increase the identification of membrane protein targets from a proteome in a native state as is required by the TPP methodologies. We evaluated the composition and distribution of protein classes in the soluble proteome extracted under native conditions with an aqueous solution of PBS containing a nonionic surfactant Igepal or NP-40S in comparison to the PBS-extracted proteome as a control. Here, we analyzed by quantitative proteomics the soluble proteome, a sample that could be further utilized for TPP, and the insoluble fraction collected in the pellet after centrifugation. The number of proteins in each hit was normalized against the total number of proteins. The number of membrane proteins solubilized in the different solutions were similar, 23% in PBS (1,550 proteins), 24% in Igepal (1,618 proteins), and 22% in NP-40S (1,403 proteins). However, looking at specific GO categories that contain integral membrane proteins, such as the leaflet of membrane bilayer and the side of membrane, NP-40S increased by 50% and Igepal by 20% in the number of these membrane proteins in the soluble fraction compared to control (Figure 1A,B).

The extraction methods barely increased the diversity of membrane proteins in the solution, although nonionic surfactants above their critical micellar concentration (CMC) were introduced in the extraction solution. Similar solubilization solutions for TPP analysis with samples from K562 cells rendered only 18% of membrane proteins in the solution.^10^ The conditions to facilitate that a membrane protein would embed in a micelle are complex and require the convergence of many more factors in addition to the surfactant concentration in the extraction solution.^21^ It could require trans-bilayer movements that could be relatedly slow.^22^ Based on the concentration of the surfactant in the solution and the marginal incorporation of membrane proteins, our solubilization results showed a low membrane protein occupancy of surfactant micelles. It implies the presence of a large proportion of empty micelles and monomers if these soluble proteomes are studied by TPP analysis. It is well-studied that surfactants bind to proteins in solution mainly by electrostatic, hydrophobic, and h-bonding.^15,23^ It could be expected that several populations of surfactants, from monomers and micelles to self-assembly aggregates, will be available for possible interaction with proteins.^15^

### TTP Analysis and Effect of the Temperature on the Alteration of Protein Solubility in Aqueous Micellar Solutions

**Figure 2:**
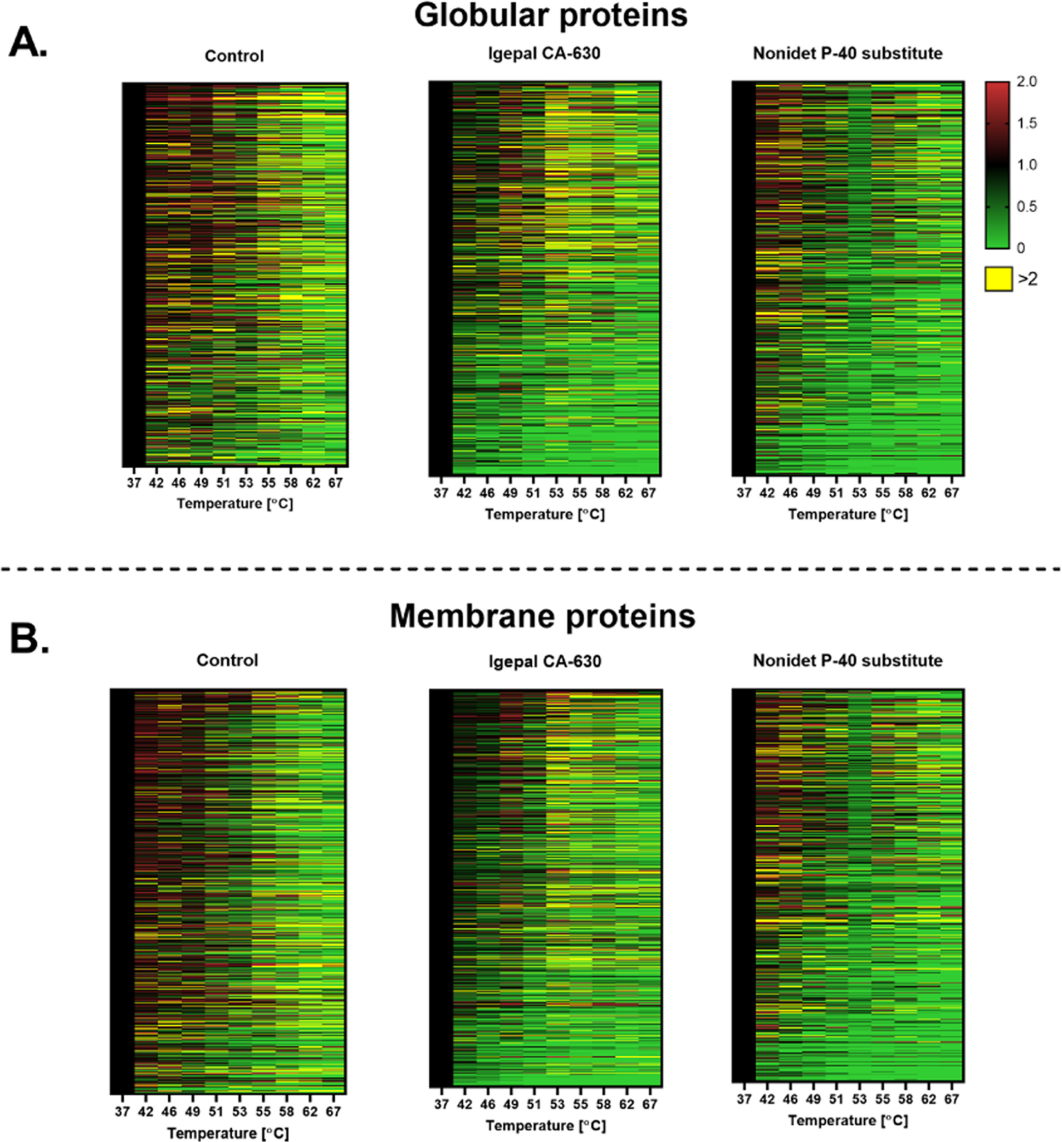
Heatmap representing the effect of the temperature on the alteration of protein solubility in aqueous micellar solutions. The soluble fractions were separately exposed to a range of temperatures as in TPP methodologies. The fractions were analyzed by quantitative mass spectrometry (LC-MS/MS). The proteins were classified by (A) globular or (B) membrane protein via UniProt using Gene Ontology classification. The *x*-axis represents the temperatures, the *y*-axis represents proteins, and each row is one protein. Proteins were normalized against the lowest temperature (37 °C). Each value is the mean of two replicates.

Analyzing TPP proteomes solubilized by the previously described conditions, the occurrence of interactions of globular or membrane protein with a different subpopulation of surfactant molecules could not be excluded based on previous results. The principle of the TPP method for the identification of targets relays on the detection of the alteration of protein stability under thermal stimulus caused by protein–chemical compound interactions.^24^ Therefore, we aimed to evaluate if the interaction between surfactants and proteins could also be detected in the TPP methodology as an alteration of protein stability in solution.^100^ First, we performed the TPP experiment and evaluated the alteration in protein solubility along the thermal shift range of temperatures in the different solubilization conditions. A total of 3,340 proteins were identified in the samples and then filtered according to the requirements for TPP analysis.^24^ In the control sample in PBS, the number of proteins identified at the lowest temperature (37 °C) was 1,436 and 1,738 and 1,608 in Igepal and NP-40S, respectively. Considering that the surfactant–protein interactions will respond to different principles for globular than membrane proteins, the analysis of alteration in solubility has been presented separately (Figure 2).

For globular proteins in the Igepal solution, proteins are destabilized at a lower temperature than control. An erratic pattern of proteins changing in solubility from 53 to 67 °C was visible on the heatmap. In the samples in NP-40S, a surfactant with a CPT at 53 °C, a minimal impact is detected at lower temperatures but a sharp decrease at CPT, 53 °C. For the membrane proteins in Igepal, the decrease in protein solubility is higher than in control along the thermal shift range. For NP-40S, similar to the globular proteins, the impact of the phase separation around the CPT temperature marked a decrease in solubility that is higher than at any other temperature of the studied range. In summary, the alteration in protein solubility of proteins in the Igepal solution followed erratic changes. In the case of NP-40S, the impact of the CPT was denoted in the sharp effect of protein destabilization at that temperature (Figure 2).

The results showed that the presence of the nonionic surfactant in the extraction solution has an overarching effect on the proteome, altering the protein solubility for both globular and membrane proteins. This effect is more prominent in the proximity of the surfactant CPT, that is, the temperature where a phase separation between high and low surfactant concentration is expected to be formed.^26^

### TPP Analysis Determines the Shift of the Protein Melting Point (Tm)

**Figure 3:**
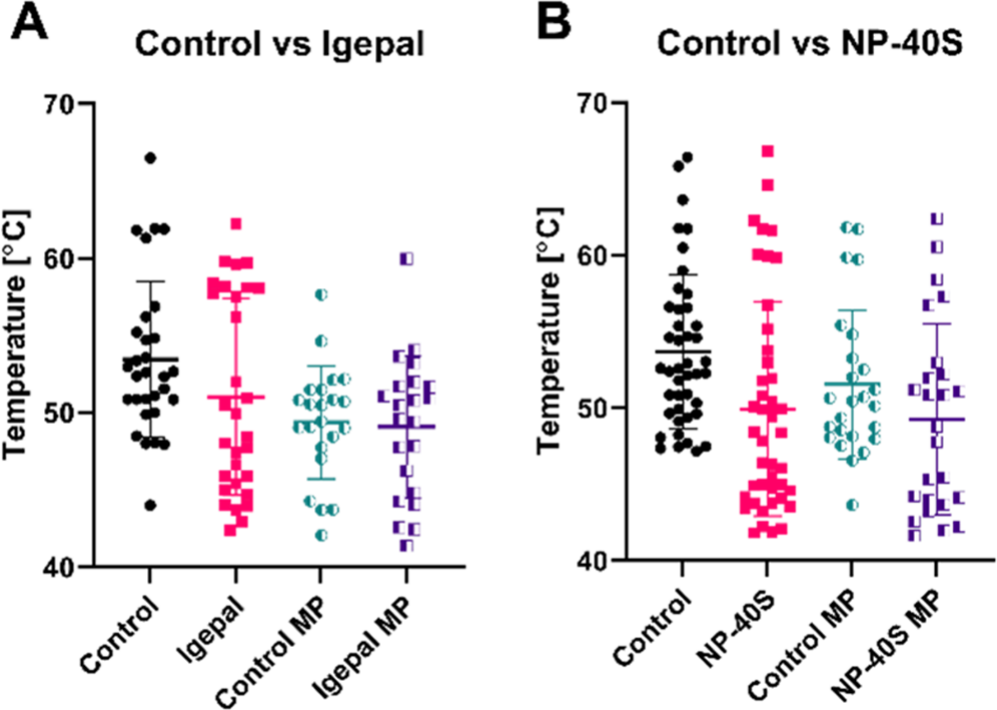
Proteins with shifted melting points (Tm). Proteins with variations in Tm were plotted with a mean melting point. (A) Control set as a vehicle for the TPP analysis and Igepal set as an interactor. (B) Control set as a vehicle and NP-40S set as an interactor. In all graphs, the middle bar is the mean value of two replicates and the error bars are the standard deviation (SD). MP = membrane proteins.

The alteration of the protein solubility observed in the previous experiment could suggest variation in the protein Tm. The alteration of the Tm is the factor used in TPP to identify protein targets. Therefore, we performed TPP analysis to detect differences in melting point, plotted in Figure 3. All comparisons started with 3,340 proteins, and melting curves were later narrowed down based on four quality criteria as described in the method.^5^ For this TPP analysis, the control was equivalent to the sample with the vehicle, and the solution with the surfactant was defined as the interactor, that is, the treatment, according to the TPP R package.^5^ The control versus Igepal analysis showed 52 proteins, including 23 membrane proteins, that had a difference in Tm (Figure 3A). In control versus NP-40S, 68 proteins, including 25 membrane proteins, have altered their Tm (Figure 3B). The membrane proteins represent 44 and 37% of the proteins with shifted Tm when the aqueous solution contains surfactant micelles. (A list of all proteins, melting points, and *p*-value can be found in the supplementary Table SI–II). When Igepal was analyzed as the interactor, the globular proteins shifted their Tm to lower temperatures than the control. The membrane proteins had a wider distribution of either increased or decreased Tm. In the case of NP-40S, both globular and membrane proteins showed lower Tm than the control.

Changes in composition and turbidity of the protein microenvironment are expected with the increase in temperature and in the proximity to CPT. The primary effect of increasing the temperature is to reduce the degree of structure of water near the micelle surface, facilitating the increases of Van der Waals attraction due to the closest contact.^27^ This attractive interaction between spherical micelles of certain sizes facilitates closer contact between micelles and leads to strong spatial changes in the microenvironment.^27^ However, it is not easy to model or predict the thermally dependent variation on protein stability in this type of combined proteome that contains globular and membrane proteins. These results showed a different pattern in the thermally dependent alteration of protein solubility for globular and membrane proteins. Both protein classes in the presence of surfactants are described to follow different principles for their stabilization in solution, and therefore, multiple factors could lead to protein–surfactant interactions.^28^

In the case of globular proteins, it should be considered if the reason for alteration in protein solubility was the interaction with a monomer, a micelle, or other surfactant self-assemblies. In some cases, such as human growth factor, individual monomers could bind to hydrophobic patches that could lead to aggregation^29^ and destabilization, but for interferon-γ, the protein is stabilized with the interaction with a surfactant.^30^ The cooperation of nonionic monomers with micelles has also been shown to promote binding to globular proteins and denaturation.^101^ Several methodologies could be applied to purified proteins to evaluate the interaction of proteins and small molecules, such as limited proteolysis, among others.^100^ However, it would not apply to a proteome-wide approach such as TPP methodologies. These results open the question of how the TPP analysis could distinguish between alterations in target solubility based on the interaction with a surfactant from alteration in solubility due to interactions with a chemical or a drug. Therefore, the applicability of the TPP principle of thermally induced alteration of protein solubility to a proteome extracted with nonionic surfactants could also compromise the robust identification of globular proteins as targets.

In the case of the membrane proteins embedded in micelles, several factors could cause thermally induced alteration in their solubility, starting from the solubilization process that included the trans-bilayer movement, a sequential process, and complexity.^22^ This process not only required micellization and the saturation of the membrane bilayer with a surfactant but also the transition of the whole bilayer to thread-like mixed micelles.^32^ The thermally induced changes in the surfactant forms described could also compromise membrane protein solubilization. Second, a membrane protein could be embedded in micelles of different sizes. The size of the micelle is a key factor in the precipitation of occupied micelles by sedimentation. The fraction of lipids from the bilayer transferred into the micelles would vary from protein to protein, and the same protein could be embedded in micelles of different sizes.^22^ The thermal shift would also alter the fluidity of lipids inside the micelle, promoting the micelle size transition.^8^ These micellar structural changes could determine the membrane protein precipitation without even any direct alteration of membrane protein stability. Third, a larger proportion of empty micelles compared to the portion of micelles embedding integral membrane proteins should be expected based on the results. There were very limited increases in membrane protein solubilization with the nonionic surfactant extraction conditions normally used in TPP.^10^ Consequently, empty micelles and occupied micelles should coexist in the solution and be available for interactions. Summarizing, it would be very difficult to determine that any observed alteration in the solubility of a membrane protein embedded in micelles is exclusively caused by a protein–chemical interaction. It should be considered that in the case of chemical–protein interaction with membrane proteins, it would take place on a minor hydrophilic domain. This type of domain frequently offers some degree of disorder and flexibility. However, the larger part of the membrane protein, the hydrophobic domain, is expected to stay protected inside a large surfactant micelle that has shown to be very sensitive to thermally induced alteration that compromises membrane protein solubility.

### Analysis of Aqueous Micellar Solution of Nonionic Surfactant Used to Solubilize Membrane Proteins for TPP Analysis

After evaluating proteomes extracted by nonionic surfactant solutions and determining a thermally dependent alteration of protein solubility, we aimed to analyze the effect of the temperature in the surfactant solutions. The Igepal and NP-40S solutions were prepared at the concentration and procedure utilized for TPP analysis that we have previously described. The DLS measurement from these surfactant solutions was performed at ten different temperatures between 37 and 67 °C, as used for TPP. The images from the DLS wells and DLS measurements showed thermally dependent changes in the size and aggregation of the surfactant in the solution.

**Figure 4:**
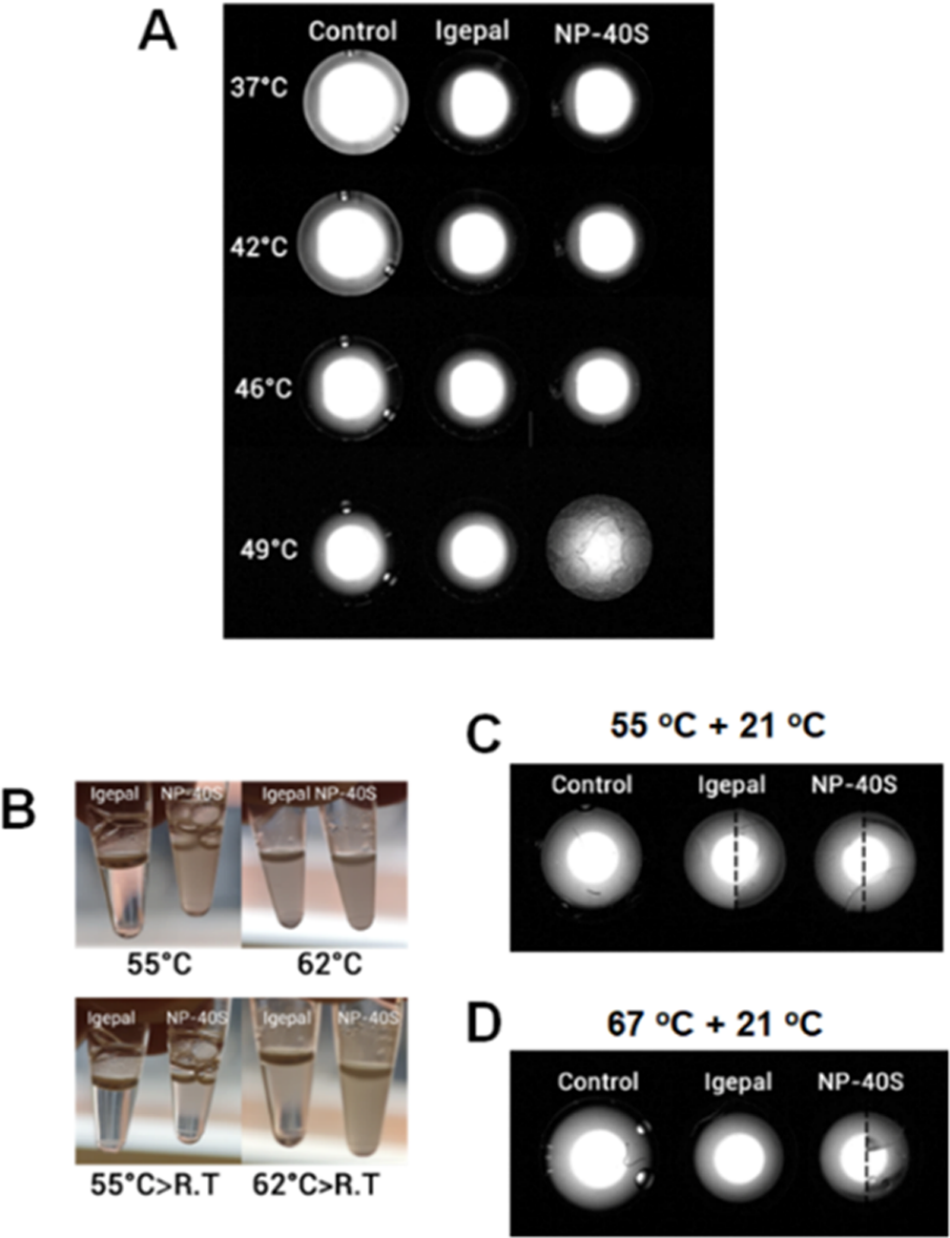
Images from thermal alteration in surfactant solutions and proteins extracted with surfactant solutions. (A) Images from the bottom of the DLS wells heated at 49 °C. Samples left-to-right, control PBS, 0.4% Igepal in PBS, and 0.4% NP-40S in PBS. (B) Images from tubes containing the soluble proteome extracted with Igepal (left) and NP-40S (right) after different thermal treatments: heating to 55 °C, heating to 62 °C, heating to 55 °C, and cooling to RT, and heating to 62 °C followed by cooling to RT. (C) Images from DLS wells control PBS versus the two surfactant solutions heated to 55 °C and then cooled to 21 °C. (D) Images from DLS wells control PBS versus the two surfactant solutions heated to 67 °C and then cooled to 21 °C. The experiments were performed with and without silicone layering to evaluate any possible effects of evaporation. The samples with longitudinal sections are composites where the left side corresponded to the experiment without silicone layering and the right with covering.

The DLS well images from the NP-40S solution at 49 °C started to show visual indications of structural changes and turbidity, but at lower temperatures, variation cannot be visually detected. The instrument could not take images at temperatures higher than 49 °C for safety reasons (Figure 4A). In addition, a different experiment simulating the heating and subsequently cooling step from the TPP protocol was performed with both the surfactant extracted proteomes (Figure 4B) and with surfactant solutions alone (Figure 4C,D). The proteomic samples were heated at 55 and 62 °C and cooled to RT. The surfactant solutions were first heated until 55 and 67 °C and cooled at 21 °C. Images from the tubes containing the soluble proteomes revealed changes in the turbidity after heating and cooling (Figure 4B). Similarly, the images from DLS wells from the surfactant solutions still showed turbidity after the cooling (Figure 4C,D).

**Figure 5:**
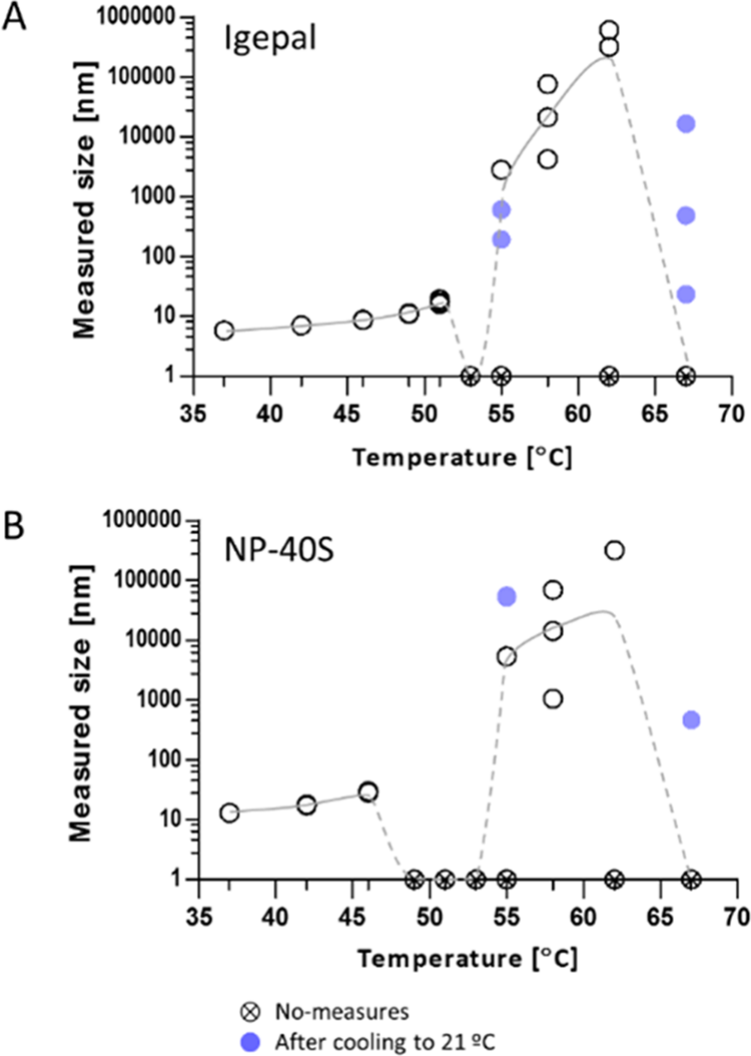
DLS measurement of the nonionic surfactant solutions exposed to 10 stepwise increases of temperature. (A) Igepal solutions exposed to 37–67 °C. (B) NP-40S solutions exposed to 37–67 °C. White circles represent DLS measurement, crossed circles represent no-measurement due to auto-attenuating error at the DLS instrument, and blue circles were DLS measurements obtained from samples that were separately heated at 55 or 67 °C (above CPTs) and cooled to 21 °C. Several data points could not be obtained due to technical reasons.

Thermally induced structural alterations of the surfactant solutions were detected from the DLS measurements. In the case of Igepal, DLS measurement from 37 to 53 °C could be correlated with the expected micellar size and its gradual increase up to the CPT. Data points were difficult to register around 53–55 °C when the solution was likely entering into clouding. The reported CPT for Igepal by the suppliers was 53–67 °C. The DLS measurement for NP-40S also suggested a thermally dependent increase in the micellar size that was expected to be slightly larger than Igepal. The missing data point between 48 and 55 °C for NP-40S due to instrumental error connected to turbidity was also close to the expected CPT around 45–50 °C. Both surfactant solutions showed a sharp increase in the DLS measured size from 55 to 65 °C, which were several orders of magnitude above a micellar size but could correspond to complex surfactant self-assembly behavior^33^ (Figure 5A,B). The DLS measurement from the heating and cooling experiments with surfactant solutions corroborated the presence of large surfactant self-assemblies that did not revert after 3 min at 21 °C (Figure 5A,B blue circles).

The expected changes in the structure of the nonionic surfactant in an aqueous solution are defined by the concentration, solution, and temperature.^16^ Our study evaluated the direct effect of the temperature by maintaining the rest of the parameters constant. The surfactant solutions at concentration and the increases in temperature used for TPP analysis showed prominent structural changes. These changes could be related to micellar growth, clouding, and the accumulation of large structures of surfactant self-assemblies and did not revert after the cooling step.

The solubility of nonionic surfactants in water has been described as a delicate balance between hydrophobic and hydrophilic interactions that assist in maintaining the surfactant molecules’ solubility. The effects of the increase in temperature can easily modify these interactions and decrease the surfactant solubility. Small changes in the effective interaction between the surfactant and water cause drastic effects such as phase formation.^33^ At clouding, the separation between the surfactant-rich and surfactant-lean starts with changes in turbidity that can be macroscopically observed.^16,33^ This is an endothermic transition attributed to the conformational rearrangement of the assembled surfactant molecules, especially the polar head groups, and its changes in the interaction with water molecules.^34^ According to the models proposed, the increase of temperature in the transition phase could lead to a removal of water molecules that were associated with the polar head groups at the bonds, breaking the intermolecular hydrogen bonds. Losing the solvated molecules may induce conformational changes in the micelles with a decrease in volume, causing a decrease in stability at the spherical shape. Therefore, the micelles could tend to adopt a more planar arrangement that would stabilize the surfactant molecules in the new environment.^16^ The reduction of the micellar curvature could also facilitate the association of micelles to micelles with the formation of long cylindrical micelles with bigger volumes. These larger and elongated micelles would require lower centrifugation force to sediment than the smaller micelles that were mainly present at lower temperatures. Therefore, the structural changes of surfactant solutions with the temperature are expected to alter the precipitation pattern of globular proteins interacting with a surfactant and membrane protein in surfactant micelles. It should be reminded that in TPP methods, the centrifugation force is applied to discriminate between soluble and insoluble proteins and to identify the chemical–target interaction.

### Microscopic Imaging of Proteins Exposed to TPP Thermal Treatment

Microscopic imaging of the proteomes exposed to heat was prepared following the TPP method to visualize microscopical changes in the aqueous micellar solution in the presence of proteins. Samples were heated to 67 °C for 3 min and imaged using light microscopy within the first 3 min, following the heating period required in TPP methodology. In the images, varying sizes of protein aggregates can be observed, as illustrated with the black arrows.

**Figure 6:**
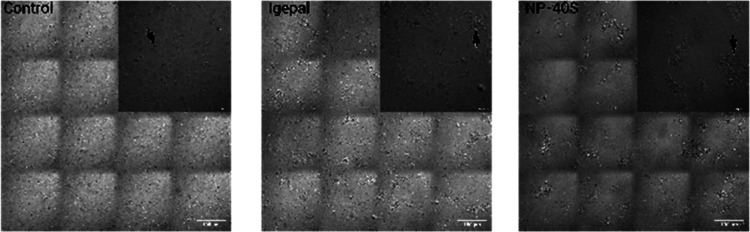
Microscopy visualization of proteins extracted with PBS (control), Igepal, or NP-40S solutions. The proteomic smaples were analyzed after following a thermal step from the TPP methodology, heated to 67 °C and visualized within the first 3 min. The largest image is merged tiles containing a scale bar of 100 μm. The smaller image is one tile with a scale bar of 30 μm. The black arrow shows one example of a protein aggregate.

The control contained small protein aggregates located close to each other, the Igepal sample contained slightly larger protein aggregates, and NP-40S contained the largest protein aggregates, which are located further apart (Figure 6). These microscopic images showing larger surfactant and protein aggregates confirm that the proteomic samples exposed to the TPP highest temperature followed a similar pattern compared to the surfactant solutions.

## Conclusions

The use of a nonionic surfactant in the proteome analyzed by TPP introduces parameters that could alter the protein solubility independently from the chemical–target interaction. We showed that the standard protocol utilized did not facilitate the solubilization of integral membrane proteins. However, soluble proteomes in surfactant solutions could contain a large proportion of empty micelles available for interaction with both globular and membrane proteins during the TPP workflow. The effects of these interactions were indirectly determined by the broad alteration in protein solubility in the studied proteomes, shift of the protein Tm, and microscopic imaging of proteins forming aggregates. The application of the TPP thermal treatment to the surfactant solution demonstrated that it followed the expected structural changes, including the increase in the micellar size and formation of other self-assembly structures at the macroscopic size. Our results lead to the exploration of thermally independent solubilization strategies to assure a confident membrane protein target identification by a high-throughput method based on proteome solubility alteration.

## Supporting Information Available

Detailed experimental protocols, including tissue protein extraction, proteomic sample preparation, nano liquid chromatography-mass spectrometry (LC-MS/MS) methods, TPP data analysis, and a list of all proteins, melting points, and *p*-values (PDF)

## Abbreviations

MS: mass spectrometry
TPP: thermal proteome profiling
NP-40S: nonyl-phenyl-poly(ethylene glycol)
Igepal: Igepal CA-630
NP-40: octyl-phenoxy(polyoxyethylene)ethanol
CPT: cloud point temperature
DLS: dynamic light scattering
Tm: melting point
TSA: thermal shift assay
