# Supplementary material for: Nonionic Surfactants can Modify the Thermal Stability of Globular and Membrane Proteins Interfering with the Thermal Proteome Profiling Principles to Identify Protein Targets
Source: Anal Chem. 2023 Feb 13;95(8):4033–42. doi: 10.1021/acs.analchem.2c04500 (PMC9979136; doi:10.1021/acs.analchem.2c04500)
Supplement: Supplementary file 1 — ac2c04500_si_001.pdf [file ac2c04500_si_001.pdf]

SUPPORTING INFORMATION

Nonionic surfactants can modify the thermal stability of globular and membrane proteins interfering with the thermal proteome profiling principles to identify protein targets

Emmanuel Berlin[1‡†], Veronica Lizano-Fallas[1‡], Ana Carrasco del Amor[1], Olatz Fresnedo[2], and Susana Cristobal[1,3*]

[1]Department of Biomedical and Clinical Sciences, Cell Biology, Faculty of Medicine, Linköping University, Linköping, 581 85, Sweden. [2] Department of Physiology, Faculty of Medicine, and Nursing, University of the Basque Country UPV/EHU, Leioa, 489 40, Spain. [3] Ikerbasque, Basque Foundation for Sciences, Department of Physiology, Faculty of Medicine, and Nursing, University of the Basque Country UPV/EHU, Leioa, 489 40, Spain.

***Corresponding Authors**

Prof. Susana Cristobal. Department of Biomedical and Clinical Sciences, Cell Biology, Faculty of Medicine, Linköping University, Linköping, 581 85, Sweden. Email: susana.cristobal@liu.se. Phone: +46-730385867.

Table of contents

Supplementary tables (pages S2-S6)
- Table S1. Differences in melting point. Control versus Igepal (page S2)
- Table S2. Differences in melting point. Control versus NP-40S (page S4)

Extended materials and methods (pages S6-S7)
- Protein extraction (page S6)
- Proteomic sample preparation by Ultrasonic-Based Filter Aided Sample Preparation (page S6)
- Nano liquid chromatography–mass spectrometry (LC-MS/MS) analysis (page S6)
- Thermal proteome profiling data analysis (page S7)

References (page S7)

Supplementary tables

1. Differences in melting point

Table S1. Control versus Igepal. Melting points of control versus Igepal were deemed to have significant differences in melting point (°C).

Protein ID	p-value	Control 1 melting point	Control 2 melting point	Igepal 1 melting point	Igepal 2 melting point	Membrane protein?
A0A0G2JSL8	0,00540185	50,7767051	50,9221086	51,9050435	51,4575308	Yes
A0A0G2JSU8	0,00796226	50,4120043	48,4553987	53,5108695	53,7539952	Yes
A0A0G2JVM0	0,00127693	46,7404893	48,7696432	42,6333378	42,5210238	Yes
A0A0G2K3K2	0,0118378	48,9456977	49,2034033	45,0669228	44,5334136	Yes
D3ZGY4	0,00510369	51,7112668	51,2800315	46,1579154	46,3498469	Yes
F1LPV8	0,01377531	49,8566479	48,1839852	51,6192578	51,793988	Yes
O88618	0,01594149	50,7567677	50,7034299	46,7246265	48,8134996	Yes
P02680	0,01223651	52,2673897	51,9809523	46,0665414	49,5868119	Yes
P08011	0,00630277	49,5921586	48,4222403	43,2053943	44,878113	Yes
P10634	0,00264906	46,8016468	50,1039276	41,0376737	43,8781869	Yes
P11711	0,04747374	41,5525948	42,603827	49,5443566	49,2567707	Yes
P16617	0,00147144	50,8511714	50,2336488	51,777674	52,1904541	Yes
P20070	0,00061777	43,0264641	44,3801745	49,8438486	51,0850389	Yes
P20673	0,02766329	52,1997995	52,186412	50,6856355	50,8913414	Yes
P34058	0,00127693	55,4970973	53,7703033	50,6390839	51,5263726	Yes
P63018	0,01849382	50,8064551	50,159342	52,2935786	54,1110465	Yes
Q09073	0,02583379	46,8296728	47,2242471	42,1852758	40,5618801	Yes
Q4KMA8	0,01272818	57,7811357	57,5346658	48,2453377	50,9787914	Yes
Q5M9H2	0,0236471	51,1988511	51,7989867	61,9524002	57,9914829	Yes
Q66HF3	0,01319786	45,1384557	42,3200999	50,8444268	50,986118	Yes
Q9WVK3	0,00060873	50,7758197	50,8359233	44,9651945	43,571371	Yes
Q9Z2L0	2,3769E-05	43,5281362	45,0325477	53,034202	55,0755031	Yes
A0A096MIX1	0,00097135	50,873583	50,8649762	43,537191	41,2811323	No
A0A0G2JSV0	0,01361306	52,6452924	54,0895422	62,0807081	57,4538862	No

A0A0G2JTL5	0,03307392	48,0544908	48,939493	43,0098813	46,4116351	No
A0A0G2JXT3	1,369E-05	51,9400496	52,7318757	45,9076529	45,9218476	No
A0A0G2QC04	0,03695056	52,9191255	54,2118377	50,7886813	50,1747662	No
B1WC26	0,00622572	57,0424206	56,6808186	57,8918605	57,0764613	No
D3Z8I7	0,00084152	52,3551251	52,3929324	43,2972847	47,5148752	No
D3ZUU8	0,00020123	55,1626323	55,2421971	50,9223233	51,0083696	No
F1LRV4	1,7851E-05	50,8262712	50,8907792	44,3741321	43,7223565	No
F1LZ34	0,02918255	50,8833406	52,2246626	61,8752089	62,6282273	No
F7EV94	0,0236471	47,3277169	48,786561	45,1060516	44,8929935	No
G3V617	0,04581213	50,965951	50,8312147	48,0058086	48,8765448	No
M3ZCQ0	0,01519224	47,2202332	48,6701561	42,8156469	44,6002379	No
P24473	0,00263864	47,0029415	49,0034302	57,5865762	54,8267688	No
P29266	0,02922103	61,3294696	61,2939244	57,9441546	58,282011	No
P32232	4,3287E-05	54,6738232	57,7490831	57,9564291	61,423994	No
P55159	0,00095654	53,5253929	51,7053998	58,284411	58,0028303	No
P57093	0,0043998	53,1558947	52,1700732	47,953255	43,7797471	No
P57113	0,00167983	48,2750335	51,7611195	44,1084026	43,8386699	No
P68255	0,0132931	62,22893	61,3901202	58,2317246	57,1534834	No
P85834	0,01377034	50,9576564	50,7873878	47,5254026	48,5493177	No
Q07071	0,00018007	43,565488	44,4454497	47,795059	47,6411667	No
Q3MID4	0,00742899	54,5962441	54,824389	45,1906124	48,0644182	No
Q3MIE0	0,02142063	54,9378376	54,7348732	50,2458865	53,7439423	No
Q4QQW3	0,03639081	61,9639016	61,8411886	61,1782283	58,0839409	No
Q5RKH2	0,0118378	49,7895186	49,9800032	45,039143	40,8903412	No
Q5XIC0	0,00147144	52,7662666	53,164995	49,5688089	50,2340613	No
Q68FR6	0,00492822	50,8418807	51,3354364	46,9493324	47,9046059	No
Q8K3R0	0,00852123	66,65881	66,3354331	58,1546209	58,0121648	No
Q8VHT6	0,01594149	61,7366091	62,0612877	58,330955	58,4931206	No

Table S2. Control versus NP-40S. Melting points of control versus NP-40S were deemed to have significant differences in melting point (°C).

Protein ID	p-value	Control 1 melting point	Control 2 melting point	NP-40S 1 melting point	NP-40S 2 melting point	Membrane protein?
A0A0G2JVM0	0,017047106	46,29792153	48,75315057	43,7061363	43,62778662	Yes
A0A0G2JYB1	0,014999614	61,88253914	61,74504132	57,01708047	57,58497837	Yes
A0A0G2K3K2	0,000513642	48,346375	49,20348645	43,1000912	45,3561162	Yes
F1LPV8	0,002765123	49,32669515	48,11897354	52,72030425	51,17620882	Yes
M0R660	0,000131941	51,93917095	50,49689924	42,62753003	42,51718079	Yes
O88618	1,37837E-06	50,42486073	50,47910635	45,6751449	45,03355049	Yes
P06761	0,000266371	59,65733217	59,78561377	62,70967309	62,09524816	Yes
P08011	2,17063E-05	48,68083528	48,40266226	41,80940632	42,70591815	Yes
P09034	0,011240123	47,43069335	48,88925816	43,48708075	44,46605629	Yes
P10634	0,004002661	46,159203	49,92652516	40,49685379	43,42945219	Yes
P12785	0,00272625	47,41420796	48,517845	41,69248944	44,80330333	Yes
P18418	0,002575991	60,1398611	59,60529956	50,31986341	54,15559576	Yes
P34058	0,001284183	56,80569617	54,05085095	51,43946921	50,3301404	Yes
P50399	0,006782931	52,73665242	53,74512939	48,19328427	49,36850891	Yes
P63018	0,001193019	50,13211867	50,1272875	54,00720281	51,93452232	Yes
P82995	0,000396041	54,53245402	55,0713913	51,03496723	51,16256522	Yes
Q09073	0,046007918	46,08601808	47,00027849	41,44754163	41,82499287	Yes
Q3MIE4	0,011179185	48,47318105	52,96179989	57,96511616	58,89736975	Yes
Q5M9H2	0,00249893	51,75487796	52,24251349	59,11159002	62,00333048	Yes
Q5XI73	0,032307044	61,56343155	61,84889672	38,77423195	56,81098475	Yes
Q64648	0,004413651	49,86567027	44,32481381	55,97854771	57,46381011	Yes
Q66HF3	0,002814444	44,98297405	42,30804797	50,96519366	50,73920364	Yes
Q66HT1	0,023481732	52,65249304	52,37363187	50,95720653	51,41739096	Yes
Q9WVJ6	0,032589104	48,41184789	50,2949107	42,21491887	46,1024215	Yes
Q9WVK3	0,000131941	50,69712056	50,6211189	45,06567944	45,83330939	Yes
A0A096MIX1	0,011134753	50,87385117	50,80795782	46,14351094	46,53592694	No
A0A0G2JTL5	0,000776464	47,52551127	48,91582266	44,10394354	43,34458062	No

A0A0G2JXT3	0,000235558	51,78987169	52,48385782	45,19889403	46,8892425	No
A0A0G2KAV5	9,83332E-05	48,31971149	50,90430672	43,18441284	43,92966811	No
B2RYW9	0,003757005	46,42610859	47,93082998	49,0292915	51,10898763	No
D3Z8I7	0,002814444	52,58547158	52,65117006	44,10287772	44,23040089	No
D3ZIC2	2,49758E-05	56,53291542	56,37427281	47,80896283	52,03802346	No
D3ZUU8	0,003037674	55,40819381	55,33624126	50,62895571	48,27511251	No
F1LZ34	0,001001435	51,58802116	52,02763241	59,34276425	60,76026413	No
G3V6C2	0,038510976	61,72907671	61,79436921	58,05876769	61,69698082	No
G3V6C4	0,013234719	48,95520521	50,89706161	42,02261946	45,51993517	No
G3V7C6	0,021009336	56,80205239	52,36344307	42,44211363	41,21150521	No
I6L9G6	0,013289534	54,36050814	51,51518003	46,16510583	46,59772971	No
M0RCU5	0,000561315	57,53680065	58,12046521	61,5288063	61,87623302	No
M3ZCQ0	0,00426238	46,35908662	48,61504167	41,25545052	42,49164092	No
P10760	0,000777629	52,07634592	53,11644193	50,61511223	50,15578767	No
P14173	2,17063E-05	61,70402461	61,75188313	55,955988	54,34296657	No
P22789	0,00023322	48,7627734	47,36055136	42,20355402	44,28608869	No
P22791	3,24917E-06	47,68863582	47,69148836	41,88230464	42,24650814	No
P30713	0,017605417	58,03797118	56,90786188	56,4921521	49,50153724	No
P41034	0,010889791	55,97371133	54,77310744	51,07079868	50,56856408	No
P48500	1,65954E-06	60,62652516	60,37039253	66,79609975	66,83049428	No
P50398	0,029768656	55,32611524	53,91314701	38,8487819	51,15009439	No
P56571	0,029768656	57,08379813	56,02859736	61,72556426	61,52610533	No
P57093	0,028748994	53,15797323	51,64072375	48,04579588	47,61728027	No
P57113	0,002995328	47,7411341	51,56288585	42,66429435	44,24960623	No
P62959	0,038763948	58,08400059	59,90079126	66,83758175	62,33999122	No
P85834	0,001196963	51,03470751	50,74461245	46,05755	43,82088089	No
Q5RKH2	0,000412364	49,01642589	49,72354926	44,66586553	44,82252847	No
Q5U2S7	0,015421117	51,37551048	53,19132354	48,32079541	48,5474676	No
Q5XIC0	0,0077387	52,70485463	53,39498371	46,87219255	49,86450291	No
Q63150	0,031854771	65,95519883	65,69099594	61,90698719	62,67279813	No

Q66X93	0,030836703	47,12837091	47,79067469	38,79032607	45,71132261	No
Q68FR6	0,02442857	50,62160659	51,03799693	43,92175722	47,10142053	No
Q68FR9	0,002698201	47,16007703	47,53036598	48,59950661	54,98711487	No
Q68FS4	0,000169691	62,88370195	64,36085909	56,49492169	56,9664055	No
Q68FU3	0,024424729	51,90832472	48,74312842	55,51050508	52,03120257	No
Q6IMY6	0,017047106	52,88976081	51,57405066	43,75078359	45,41746837	No
Q6P6U2	0,006628626	48,30835578	49,95750336	42,10987539	46,07245855	No
Q7TPB1	0,027899554	54,71083199	54,68304761	51,94216677	51,91327665	No
Q8K3R0	0,033484274	66,38877112	66,45035119	58,05955731	61,80047385	No
Q920F5	0,001294746	57,71563646	55,51313463	38,9090836	51,06798976	No
Q9ER34	0,032307044	54,02142335	54,82277592	49,43769876	50,39161657	No

Extended materials and methods

Protein extraction. Liver tissue was resuspended in buffer containing PBS (control), 0.4 % (v/v) Igepal, or 0.4 % (v/v) NP-40S in PBS. Tissue samples were mechanically homogenized using a TissueLyser (Qiagen) during 3 min at 25 Hz. Previously, zirconium oxide beads were added to each sample at a ratio of 1:1 (v/v). Subsequently, the unbroken cells were lysed by sonication in cycles of 10s/5s for 3 min at 6–10 µm amplitude at 50% intensity from an exponential ultrasonic horn of 3 mm in a Soniprep 150 MSE (MSE Ltd., Lower Sydenham, London, UK). The insoluble fraction was sedimented by centrifugation at 100,000g for 60 min at 4 °C[1]. The soluble proteome was used to perform the Thermal proteome profiling assay. The pellets were lysed with 100 µl of RIPA buffer (RIPA Lysis Buffer, BOSTER) to perform protein identification and quantification of the insoluble fraction. Protein concentration was determined by BCA assay[2].

Proteomic sample preparation by Ultrasonic-Based Filter Aided Sample Preparation. All the samples analyzed by MS were digested following this method[3]. Briefly, the proteins were reduced by adding 200 µL of 50 mM dithiothreitol (DTT) prepared in 8 M urea and 25 mM ammonium bicarbonate (AmBic) in 30 kDa microcon centrifugal filter units. Ultrasound energy was then applied using the ultrasonicator Q700 Sonicator with cup horn (Qsonica L.L.C) for 5.25 min (7 cycles: 30 s on and 15 s off UT, 25% UA, 20 kHz UF). Afterward, centrifugation for 20 min at 14 000 g was done, followed by protein alkylation by the addition of 100 µL of 50 mM iodoacetamide (IAA) prepared in 8 M urea and 25 mM AmBic solution. The alkylation step was sped up using the ultrasonicator Q700 Sonicator with cup horn for 5.25 min (7 cycles: 30 s on, and 15 s off UT, 25% UA, 20 kHz UF). Finally, 100 µL of 1:30 trypsin in 12.5 mM AmBic solution was added, and the protein digestion was processed using the ultrasonic microplate horn assembly for 5.25 min (7 cycles: 30 s on, and 15 s off UT, 25% UA, 20 kHz UF). To ensure that all the peptides were extracted, 100 µL of 3% (v/v) acetonitrile (ACN) and 0.1% (v/v) formic acid (FA) were added followed by centrifugation of 20 min at 14 000 g. This previous step was repeated one more time. The samples were acidified with 10% FA to achieve a pH between 3 and 2. The desalting process was performed by reverse phase chromatography in C18 top tips using ACN (60% v/v) with FA (0.1% v/v) for elution, and vacuum dried to be stored at -80 °C till further analysis.

Nano liquid chromatography–mass spectrometry (LC-MS/MS) analysis. The desalted peptides were reconstituted with 0.1% FA in ultra-pure milli-Q water and the concentration was measured using a Nanodrop (Thermo Scientific). Peptides were analyzed in a QExactive quadrupole orbitrap mass spectrometer (Thermo Scientific). Samples were separated using an EASY nLC 1200 system (Thermo Scientific) and tryptic peptides were injected into a pre-column (Acclaim PepMap 100 Å, 75 um × 2 cm) and peptide separation was performed using an EASY-Spray C18 reversed-phase nano LC column (PepMap RSLC C18, 2 um, 100 Å, 75 um × 25 cm). A linear gradient of 6 to 28% buffer B (0.1% FA in ACN) against

buffer A (0.1% FA in water) was applied for a period of 78 min, followed by 40% buffer B against buffer A till 95 min. The last 5 min (95-100 min) had a cleanse period with 100% B solution. The linear gradient was carried out with a constant flow rate of 300 nL/min. Full scan MS spectra were recorded in the positive mode electrospray ionization with an ion spray voltage power frequency (pf) of 1.9 kV (kV), a radio frequency lens voltage of 60, and a capillary temperature of 275 °C, at a resolution of 30,000 and top 15 intense ions were selected for MS/MS under an isolation width of 1.2 m/z units. The MS/MS scans with higher energy collision dissociation fragmentation at normalized collision energy of 27 % to fragment the ions in the collision induced dissociation mode.

Thermal proteome profiling data analysis. Melting curves were calculated using a sigmoidal fitting approach with the R package TPP [4]. The melting curves were fitted after normalization following the equation described [4,5] computed in R: $\boldsymbol{f(T)} = \frac{\boldsymbol{1-plateau}}{\boldsymbol{1+e^{-\left(\frac{a}{T}-b\right)}}} + \boldsymbol{plateau}$

where T is the temperature, and a, b and "plateau" are constants. The value of f(T) at the lowest temperature Tmin was fixed at 1. The melting point of a protein is defined as the temperature Tm at which half of the amount of the protein has been denatured. The quality criteria for filtering the sigmoidal melting curves were: (i) fitted curves for both vehicle- and compound-treated conditions had an R^2 of >0.8; (ii) the vehicle curve had a plateau of <0.3; (iii) the melting point differences under both the control and the treatment conditions were greater than the melting point difference between the two controls; and (iv) in each biological replicate, the steepest slope of the protein melting curve in the paired set of vehicle- and compound-treated conditions was below −0.06. The NPARC of the R package was used to detect significant changes in the temperature-dependent melting behaviour of each protein due to changes in experimental conditions[4]. The significance threshold was set at $p < 0.05$.

References

(1) Carrasco Del Amor, A.; Freitas, S.; Urbatzka, R.; Fresnedo, O.; Cristobal, S. Application of Bioactive Thermal Proteome Profiling to Decipher the Mechanism of Action of the Lipid Lowering 13(2)-Hydroxy-pheophytin Isolated from a Marine Cyanobacteria. *Mar Drugs* **2019**, *17* (6), 371. DOI: 10.3390/md17060371.

(2) Smith, P. K.; Krohn, R. I.; Hermanson, G. T.; Mallia, A. K.; Gartner, F. H.; Provenzano, M. D.; Fujimoto, E. K.; Goeke, N. M.; Olson, B. J.; Klenk, D. C. Measurement of protein using bicinchoninic acid. *Anal. Biochem.* **1985**, *150* (1), 76-85.

(3) Carvalho, L. B.; Capelo-Martinez, J. L.; Lodeiro, C.; Wisniewski, J. R.; Santos, H. M. Ultrasonic-Based Filter Aided Sample Preparation as the General Method to Sample Preparation in Proteomics. *Anal Chem* **2020**, *92* (13), 9164-9171. DOI: 10.1021/acs.analchem.0c01470.

(4) Franken, H.; Mathieson, T.; Childs, D.; Sweetman, G. M.; Werner, T.; Togel, I.; Doce, C.; Gade, S.; Bantscheff, M.; Drewes, G.; et al. Thermal proteome profiling for unbiased identification of direct and indirect drug targets using multiplexed quantitative mass spectrometry. *Nat Protoc* **2015**, *10* (10), 1567-1593. DOI: 10.1038/nprot.2015.101.

(5) Savitski, M. M.; Reinhard, F. B.; Franken, H.; Werner, T.; Savitski, M. F.; Eberhard, D.; Martinez Molina, D.; Jafari, R.; Dovega, R. B.; Klaeger, S.; et al. Tracking cancer drugs in living cells by thermal profiling of the proteome. *Science* **2014**, *346* (6205), 1255784. DOI: 10.1126/science.1255784.
